# A multi-center validation study on the discrimination of *Legionella pneumophila* sg.1, *Legionella pneumophila* sg. 2-15 and *Legionella* non-*pneumophila* isolates from water by FT-IR spectroscopy

**DOI:** 10.3389/fmicb.2023.1150942

**Published:** 2023-04-13

**Authors:** Alessandra Tata, Filippo Marzoli, Miriam Cordovana, Alessia Tiengo, Carmela Zacometti, Andrea Massaro, Lisa Barco, Simone Belluco, Roberto Piro

**Affiliations:** ^1^Laboratorio di Chimica Sperimentale, Istituto Zooprofilattico Sperimentale delle Venezie, Vicenza, Italy; ^2^Department of Food Safety, Istituto Zooprofilattico Sperimentale delle Venezie, Legnaro, Italy; ^3^Bruker Daltonics GmbH & Co. KG, Bremen, Germany; ^4^OIE Italian Reference Laboratory for Salmonella, Istituto Zooprofilattico Sperimentale delle Venezie, Padova, Italy

**Keywords:** *Legionella pneumophila* sg.1, *Legionella pneumophila* sg. 2-15, FTIR – Fourier transform infrared spectroscopy, *Legionella* non-*pneumophila*, validation, SVM – support vector machine, machine learning

## Abstract

This study developed and validated a method, based on the coupling of Fourier-transform infrared spectroscopy (FT-IR) and machine learning, for the automated serotyping of *Legionella pneumophila* serogroup 1, *Legionella pneumophila* serogroups 2-15 as well as their successful discrimination from *Legionella* non-*pneumophila*. As *Legionella* presents significant intra- and inter-species heterogeneities, careful data validation strategies were applied to minimize late-stage performance variations of the method across a large microbial population. A total of 244 isolates were analyzed. In details, the method was validated with a multi-centric approach with isolates from Italian thermal and drinking water (*n* = 82) as well as with samples from German, Italian, French, and British collections (*n* = 162). Specifically, robustness of the method was verified over the time-span of 1 year with multiple operators and two different FT-IR instruments located in Italy and Germany. Moreover, different production procedures for the solid culture medium (in-house or commercial) and different culture conditions (with and without 2.5% CO_2_) were tested. The method achieved an overall accuracy of 100, 98.5, and 93.9% on the Italian test set of *Legionella*, an independent batch of *Legionella* from multiple European culture collections, and an extra set of rare *Legionella* non-*pneumophila,* respectively.

## 1. Introduction

*Legionella* are Gram-negative bacteria distributed ubiquitously in natural water environments and as inhabitants in artificial water systems (Fields et al., 2002). Although more than 66 species of *Legionella* have been described, among which several were found to be related to human infections, *Legionella pneumophila* (*L. pneumophila*) is the most clinically relevant and investigated species. *Legionella pneumophila* is the causative agent of Legionnaires’ disease and Pontiac fever (Newton et al., 2010). It is an opportunistic pathogen of public health concern, especially for more susceptible people (e.g., elderly adults, smokers or people with weakened immune systems), who are particularly prone to the infection and at risk of developing clinical complications and respiratory failure. *Legionella pneumophila* is characterized by 16 different serogroups, with serogroup 1 (sg.1) being the most clinically relevant (Ciesielski et al., 1986). *Legionella pneumophila* sg.1 accounts for 80–90% of Legionnaires’ disease cases (Legionnaires’ disease – Annual Epidemiological Report for 2020, 2022). The other serogroups only occasionally cause legionellosis. The European Legionnaires’ disease Surveillance Network (ELDSNet) regularly monitors the outbreaks of Legionnaires’ disease in Europe. On October 2021, ELDSNet published the results of the 2020 surveillance, reporting a notification rate of 1.9 cases per 100,000 population for the Europe and European economic area (EU/EEA) with four countries (France, Germany, Italy, and Spain) accounting for 72% of all cases (Legionnaires’ disease – Annual Epidemiological Report for 2020, 2022). It is worth noticing that these pneumonia-causing bacteria spread their habitat range with climate change and the increase of the temperatures and humidity (Mirsaeidi et al., 2016).

Currently, *Legionella* control plans are mandatory for thermal baths, hotels and public structures, cooling towers, water tanks, pools, spa, and fountains. However, the European Parliament revised the Drinking Water Directive (DWD) (Directive EU 2020/2184 of the European parliament and the Council of 16 December 2020 on the quality of water intended for human consumption, 2020) to (i) improve water’s safety parameters, (ii) increase the citizens’ trust of tap water, and (iii) encourage tap water use with the aim of reducing the use of the plastic bottles. For these reasons, the revised DWD also requires *Legionella* monitoring in all drinking water distribution systems in Europe. The revised DWD entered into force in January 2021, but the EU member states must transpose it in their national legislation (and thus become compliant) by January 2023. Therefore, robust and fast methods will soon be necessary to detect *Legionella* in a large amount of potable water samples. The reference method used for the culture and the quantification of environmental samples of *Legionella* are described in the ISO 11731:2017 (n.d.) (“ISO 11731:2017. Water Quality —Enumeration of Legionella”). The confirmation of serogroup for *L. pneumophila* isolates has clinical significance and epidemiological value and is recommended by Italian national guidelines (Linee Guida per la Prevenzione ed. il Controllo della Legionellosi, 2015). It is usually carried out by immune-chromatographic and agglutination tests (Zähringer et al., 1995; Helbig et al., 1997). Besides these, polymerase chain reaction (PCR) tests can be applied (Gaia et al., 2005; Ratzow et al., 2007). The identification of *L. pneumophila* serogroups and *L.* non-*pneumophila* could be burdensome for the laboratories due to the costs of immune-chromatographic and multiplex real-time PCR assay. For this reason, spectrometric methods have been established in recent years for species identification and for the serotyping of *L. pneumophila* (Gaia et al., 2011; Dilger et al., 2016; Trnková et al., 2018; Kyritsi et al., 2020; Pascale et al., 2020; Blanco et al., 2021; Tata et al., 2022).

In the present study, a Fourier-transform infrared spectroscopy (FT-IR)-based method, coupled to a linear support vector machine (SVM), was developed, optimized, and validated for discrimination of *L. pneumophila* sg.1, *L. pneumophila* sg.2-15, and *L.* non-*pneumophila*. The principle of the FT-IR spectroscopy is based on the absorption of the infrared (IR) light by the whole bacterial cells. The absorption of the IR radiation causes the excitation and vibration of a variety of macromolecules of the cell such as polysaccharides, lipids, proteins, and nucleic acids (Naumann et al., 1991). Since the different functional groups of these compounds absorb IR light at different wavenumber ranges, FT-IR spectroscopy generates infrared absorption spectra that turn in a highly specific fingerprint of each microorganism (Novais et al., 2019). In the past, FT-IR spectroscopy was already proposed for the screening of both Gram-positive and Gram-negative bacteria including *Listeria monocytogenes*, *Streptococcus pneumoniae*, and *Salmonella* in clinical and food fields (Kim et al., 2005; Davis and Mauer, 2011; Cordovana et al., 2021; Deidda et al., 2021; Cordovana et al., 2022; Pascale et al., 2022; Passaris et al., 2022). The technique showed good potentials in the serotyping of *Escherichia coli* (Beutin et al., 2007; Mora et al., 2011)*, Yersinia enterocolitica* (Kuhm et al., 2009)*, Staphylococcus aureus* (Grunert et al., 2013) and *L. pneumophila* (Pascale et al., 2022). Recently, FT-IR spectroscopy was employed also for the identification of bacteria responsible for outbreaks in hospitals (*Pseudomonas aeruginosa*, *Klebsiella pneumoniae*, *Enterobacter cloacae*, *Acinetobacter baumannii*) or in the community (*S. pneumoniae*), demonstrating also its role as fast and cost-effective method for the implementation of infection control measures (Martak et al., 2019; Passaris et al., 2022; Wang-Wang et al., 2022). Note that the combination of FT-IR spectroscopy with machine learning, in the microbiology field, experienced a significant horizontal growth throughout the last two decades, with no single method having broken through the accreditation barrier due to lack of proper validation. In this context, a previous proof-of-concept study demonstated the capability of FT-IR spectroscopy of discriminating each *L. pneumophila* serogroup and differentiating *L. pneumophila* sg.1 from *L. pneumophila* sg. 2-15 with an explorative approach (Pascale et al., 2022). On the contrary, the present study developed and fully validated a method, based on the coupling of FT-IR and machine learning, for the automated differentiation of *L. pneumophila* sg.1 and sg. 2-15, as well as their discrimination from *Legionella* non-*pneumophila.* Including isolates from different European collections, our study describes the challenges associated with a large microbial population and evaluated robustness of the method over an extended period of time with multiple operators, FT-IR instruments and slightly different culture conditions. As recommended by the sole non-targeted methods guidelines (USP Pharmacopeia, 2018), the robustness of the method was established in terms of accuracy, sensitivity and specificity and then monitored with an extended validation approach.

## 2. Materials and methods

### 2.1. Samples

A total of *n* = 244 *Legionella* isolates were cultured and analyzed by FT-IR coupled to a Biotyper® system, briefly named IRBT (Bruker Daltonics GmbH & Co. KG, Bremen. Germany). Specifically, the dataset included *n* = 167 *L. pneumophila* and *n* = 77 *L.* non-*pneumophila*.

Initially, *n* = 82 strains, isolated from Italian thermal and drinking water of the Triveneto region during routine analyses (October 2021–February 2022) by the food safety laboratory of Istituto Zooprofilattico Sperimentale delle Venezie (IZSVe) or retrieved from a collection of reference strains held at the hospital *Amedeo di Savoia* of Turin (Italy), were investigated. Water samples were submitted to membrane filtration and diluted on selective agar medium following ISO 11731:2017. The samples were cultured in buffered charcoal yeast extract (BCYE) agar (produced in house according to ISO 11731:2017) for 48 ± 2 h at 37°C in humid environment without controlling the CO_2_ level. *Legionella* confirmation was carried out by Virapid® *Legionella* culture immunochromatographic test (Vircell S.L., Granada, Spain) or *Legionella*-Latex test agglutination test (Oxoid, United Kingdom). These Italian samples were used for training (*n* = 18) and the first testing of the classifier (*n* = 64). Additional strains (*n* = 162), isolated from both clinical and water samples, were obtained from (i) routine analysis samples of domestic and water systems at the as well as cooling towers,at the Chemisches und Veterinäruntersuchungsamt Stuttgart (CVUAS, Germany), (ii) routine analysis of samples of domestic and water systems at the Krankenhaus der Barmherzigen Schwestern in Ried’s hospital (KBS, Austria), (iii) the German Collection of Microorganisms and Cell Cultures of the Leibniz-Institute (DSMZ, Germany), (iv) the Collection of the Pasteur Institute (CIP, France), (v) the Culture Collection University of Gothenburg (CCUG, Germany), and (vi) the National Collection of Type Cultures of the United Kingdom Health Security Agency (NCTC, United Kingdom). These samples, provided by the long-established collections of reference strains in Europe mentioned above, were used to validate the method over the time-span of 1 year. To this aim, they were cultured in the bacteriology laboratory of Bruker Daltonics (Bremen, Germany) in BCYE agar (Becton, Dickinson and Company, Sparks, MD, United States) for 48 ± 2 h at 37°C with 2.5% CO_2_ in a humid environment. Furthermore, before FT-IR analysis, these samples were further identified by 16 s sequencing at Bruker laboratories and then serotyped for serogroup by using the following agglutination tests purchased from Pro-Lab Diagnostics (Richmond Hill, Canada): Prolex™ *L. pneumophila* sg.1 Latex Monoclonal Reagent and Prolex™ *L. pneumophila* sg. 2-15 Latex polyclonal reagents.

### 2.2. Spectra acquisition and analysis

**Figure 1:**
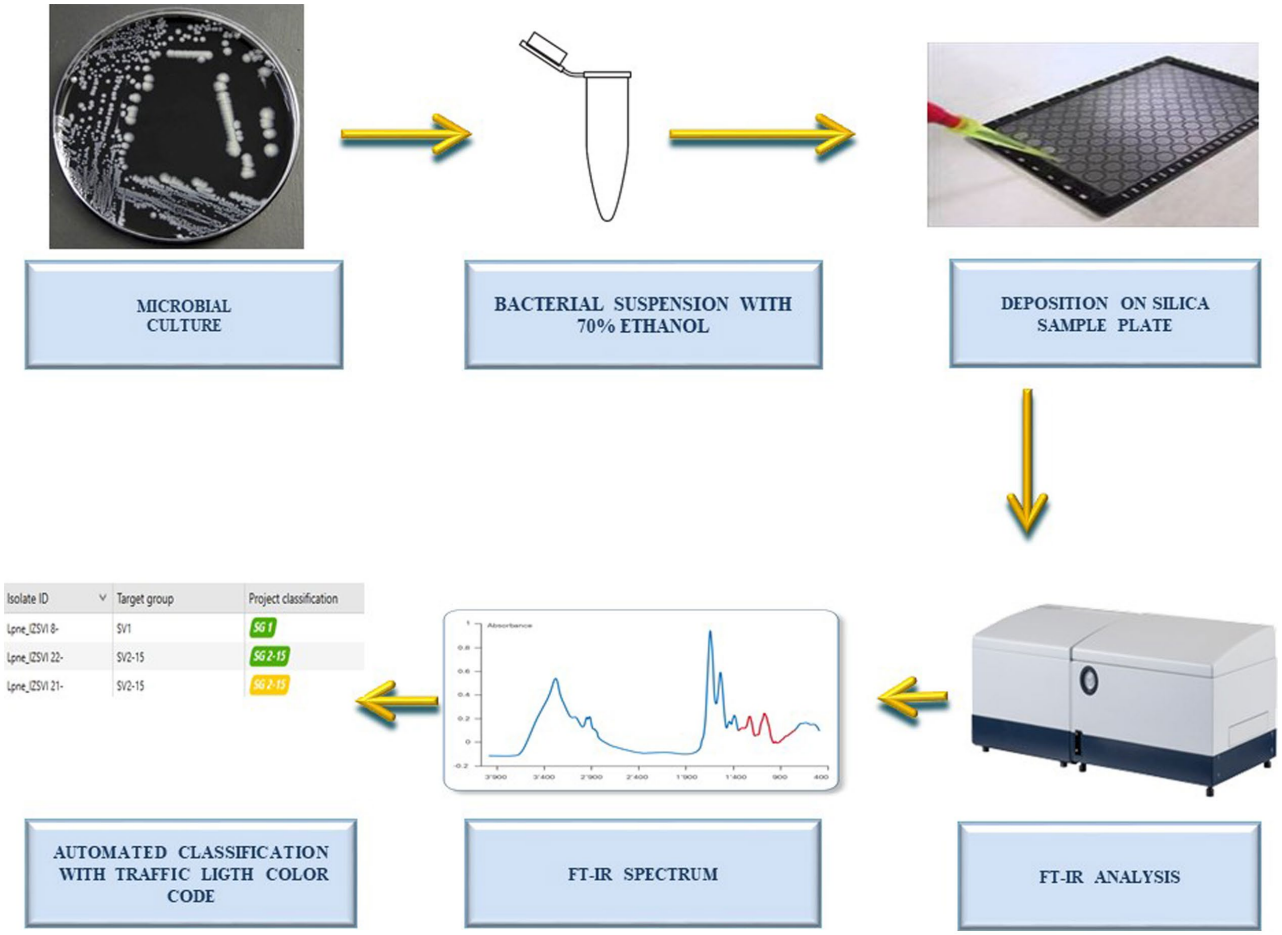
Workflow of the analysis. Workflow illustrating the steps followed by the operator for the automated serotyping of water-derived *Legionella pneumophila* sg.1, *Legionella pneumophila* sg. 2-15, and *Legionella* non-*pneumophila* by Fourier-transform infrared spectroscopy (FT-IR) and machine learning. Note that analytical time of FT-IR analysis is around 2.5 h for 96 spots (from colony picking to spectra acquisition). The classification can be performed in real time (during spectra acquisition) or retrospectively, and it takes few seconds (for hundreds samples).

IRBT spectra were acquired in two laboratory centers: (i) the Bruker Daltonics bacteriology laboratory in Bremen, Germany (*n* = 162 isolates) and (ii) the Istituto Zooprofilattico Sperimentale delle Venezie, Italy (*n* = 82). The sample preparation for IRBT measurement was performed following the manufacturer’s instructions. Briefly, a bacterial suspension was prepared in dedicated tubes contained in the IRBT kit, by suspending an abundant 1 μL loop of bacterial culture in 50 μl of 70% ethanol and vortexing to homogenize the sample. After adding 50 μL of sterile water and vortexing again, 15 μL of bacterial suspension were pipetted (in three technical replicates) on the IRBT silicon sample plate and dried at room temperature for 20–30 min. Spectra acquisition was performed with IRBT spectrometers and OPUS software (Bruker Optics GmbH & Co. KG) in transmission mode. The spectra were acquired in the spectral range of 4,000–500 cm^−1^ (mid-IR). The quality control samples IRTS 1 and IRTS 2 were analyzed in duplicate in each run. All spectra were acquired by alternating a background spectrum acquisition between each sample and quality control measurement. Processing and visualization of spectra were performed with the IR Biotyper Client Software (Bruker Daltonics, version V4.0) in the spectral region between 1,300–800 cm^−1^ (corresponding to the absorption region for carbohydrates). After spectrum smoothing using the Savitzky–Golay algorithm over nine data points, the second derivative of each spectrum was calculated, and vector normalization was applied. The workflow of the analytical method is illustrated in Figure 1.

### 2.3. Exploratory unsupervised and supervised multivariate analysis

The data analysis was performed in Bremen, Germany at the Bruker Daltonics bacteriology laboratory. Initially, an unsupervised principal components analysis (PCA) was performed on the whole dataset of isolates measured in Italy (*n* = 82) to retrieve the best principal components (PC). Unsupervised algorithms discover hidden patterns in data based on their similarities without the need of sample labeling. Unsupervised learning models are used for three main tasks: clustering, association and dimensionality reduction. The best 30 PC were used to built-up a supervised linear discriminant analysis (LDA) model. A supervised multivariate analysis, requiring a prior knowledge of the sample labeling, allows the clustering and classification of data. This combined approach provided a first investigation of the clustering capability and the discriminatory power of the FT-IR method for the three groups included in the study (*L. pneumophila* sg.1, *L. pneumophila* sg.2-15, and *L. non-pneumophila*).

### 2.4. Machine learning and development of automated classifiers

The classifier for the prediction of *L. pneumophila* sg.1, *L. pneumophila* sg. 2-15, and *L.* non-*pneumophila* was built using the SVM algorithm included in the IR Biotyper® software 4.0. As mentioned above, the SVM classifier was built-up with a subset of 18 randomly selected Italian isolates (*L. pneumophila* sg.1 (*n* = 4); *L. pneumophila* sg. 2-15 (*n* = 4); *L.* non-*pneumophila* (*n* = 10) belonging to 10 different species of reference strains, see Supplementary Table S1 for details), applying the first 20 principal components (PCs) of the PCA. The classifier was first validated on the withheld test set (*n* = 64) of Italian samples analyzed at the IZSVe laboratory. Afterwards, an inter-laboratory validation of the classifier was carried out, using French, German, British, and Austrian samples (*n* = 129) measured at Bruker’s laboratories. Finally, additional *L.* non-*pneumophila* isolates (*n* = 33), belonging to species not included in the training set of the classifier, were submitted to the classifier to further evaluate its performances and robustness. The IRBT classification result is delivered with a “traffic light” color code scoring system, which indicates the reliability of the classification, based on the spectral distance to the isolates included in the training set. The threshold values that define the reliability ranges are extrapolated by the distribution of the distance values of the validation cohort of samples considering the Youden index. The Youden index is a summary measurement of the receiver operating characteristic (ROC) curve for the accuracy of a diagnostic test (Youden, 1950). Youden index is calculated as follows: (sensitivity + specificity) -1. A “green score” means that the result is highly reliable. A “yellow score” indicates that the result of the prediction is moderately reliable. A “red score” value means that the prediction cannot be considered reliable, as the isolate spectra are located in the spectral space far from the samples included in the training set, and therefore, they could either not belong to any known class included in the training set, or the sample shows a very high technical or biological variance. The performance of the classifier was evaluated in terms of accuracy. Accuracy was defined as the number of isolates correctly classified (green and yellow scores) out of the total number of isolates. Error rate was defined as number of isolates erroneously classified (misclassification, green and yellow) out of the total number of isolates. Failed classification rate was defined as the number of isolates delivering a “red” result out of the total number of isolates. The performance of a classifier was also expressed in terms of sensitivity rate and specificity rate. The sensitivity of a classifier is defined as: sensitivity = True positives/(True positives + False negatives). On the other hand, the specificity rate is calculated as: specificity = True negatives/(True negatives + False positives). The calculations of sensitivity and specificity were applied to all three classes individually in each confusion matrix.

## 3. Results

### 3.1. Exploratory multivariate analysis

**Figure 2:**
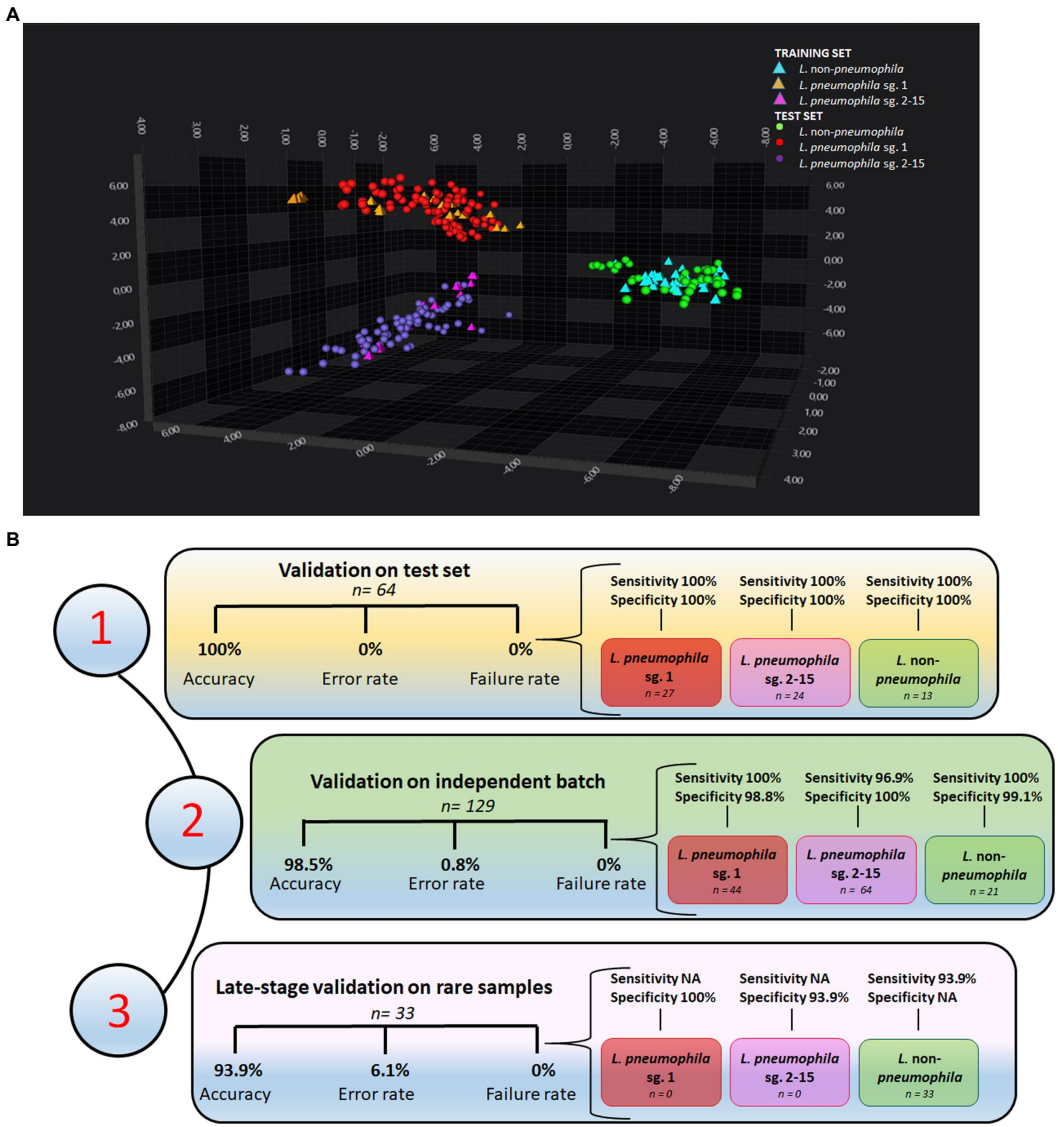
Performances of the machine learning classifier **(A)** 3D scatter plot showing the clustering of *L. pneumophila* sg. 1, *L. pneumophila* sg. 2-15, and *L.* non-*pneumophila* in the IR spectral space by PCA-LDA. The first three PC axes are shown in the diagram. **(B)** Performances of the support vector machine (SVM) classifier on (1) a test set of 64 isolates from Italian drinking and thermal waters, (2) an independent batch of samples from European collections (*n* = 129), and (3) a set of rare *L.* non-*pneumophila* from European collections (*n* = 33).

Initially, 82 isolates were analyzed by IRBT in Italy and the spectra (in triplicate) were submitted to an exploratory PCA-LDA. Altogether, 30 principal components (PC) explained 99.4% of the variance. The score plot reported in Figure 2A was generated using only the first three PCs (X axis PC1, Y axis PC2, Z axis PC3). The PCA-LDA score plot showed good clustering of the three different groups of *Legionella*, with clear differentiation of *L. pneumophila* sg.1, *L. pneumophila* sg 2-15, and *L. non-pneumophila*.

### 3.2. Performance of the machine learning classifier

A machine learning classifier, based on SVM algorithm, was initially built-up with 18 samples randomly selected among *Legionella* isolated and analyzed at IZSVe (Supplementary Table S1) and then tested on the withheld test set (*n* = 64 Italian isolates). All strains were correctly classified (Supplementary Table S2). Figure 2B shows the performances of the classifier that achieved 100% overall accuracy with 0 rate error and failure rate. Excellent sensitivity and specificity in the three groups of study was achieved.

An independent batch of *Legionella* (*n* = 129), from different European collections of reference strains, was cultured in a different laboratory with different laboratory equipment and staff, and analyzed under slightly varying culture conditions. A total of 127/129 isolates were correctly classified. Notably, *L. pneumophila* sg. 7 was misclassified as *L. pneumophila* sg.1 (Supplementary Table S3). Issues with the misclassification between sg.7 and *L. pneumophila* sg.1 were already encountered in a previous study (Pascale et al., 2022). The classifier achieved an overall accuracy of 98.5%, an error rate equal to 0.8, a failure classification rate of 0 (Figure 2B), and very good sensitivity and specificity for the three *Legionella* groups in the study.

Afterwards, the machine learning classifier was challenged with rare *L.* non*-pneumophila* isolates, retrieved from *European* collections. This further validation set comprised unusual *L.* non*-pneumophila* isolates (*n* = 33) belonging to species not included in the training set. A total of 31/33 were correctly classified, with only the *L. cardiaca* and *L. oakridgensis* isolates misclassified as *L. pneumophila* sg. 2-15 (Supplementary Table S4). *L. cardiaca* and *L. oakridgiensis* FT-IR spectra were located far away from all the other Legionella spp. strains in the spectra space. While *L. cardiaca* is a newly discovered *Legionella* species (Pearce et al., 2012) whose lipopolysaccharides (LPS) composition is still unknown, *L. oakridgensis* is less than 25% related to other *Legionella* species (Brenner, 1987) and presents only a few PLS with low molecular weight between 14 and 30 kDa (Jürgens and Fehrenbach, 1997). An overall accuracy of 93.9%, an error rate equal to 6.1, and a failure classification rate of 0 (Figure 2B) were calculated. For this group of rare *Legionella* isolates very good specificity were achieved (Figure 2B).

Finally, the combination of FT-IR spectroscopy and machine learning was inspected for its ability to differentiate *Legionella* spp. from other genera with similar colony morphology (*Bordetella* spp.*, Pasteurella* spp. and *Francisella* spp.) growing on BCYE with same conditions. This exploratory analysis, done on a small set of samples, showed that these three bacterial genera have a spectral signature very different from that of *Legionella* spp. with the consequent red score (with high outlier values) of the classifier (data not shown –further investigation are necessary to consolidate this finding).

## 4. Discussion

In this study, FT-IR spectroscopy coupled to machine learning showed excellent performances in rapid discrimination of *L. pneumophila* sg. 1 and sg. 2-15 as well as good capability of identifying *Legionella* non-*pneumophila*. Our extensive validation strategy demonstrated the robustness of our method, and its successful outcome should open new avenues through its possible adoption in routine analysis. Although the ISO 11731:2017, that regulates the enumeration of *Legionella* in water samples, does not specifically recommend the use of instrumental techniques (no other colony confirmation other than BCYE and blood agar plate growth are suggested) (ISO 11731:2017. Water quality —Enumeration of Legionella, n.d.), the present novel, non-targeted method fully satisfies this ISO guideline. On the other hand, the serotyping of *L. pneumophila* sg.1 has epidemiological and clinical values, as it allows the dissemination of this pathological serogroup and its ecological niche to be tracked and will greatly support monitoring for nosocomial infections. Currently, *L. pneumophila* serogrouping is performed using the latex agglutination test, which consists of specific polyclonal antibodies available to identify the two major groups (sg.1 and sg.2-15). Although this technique gives rapid results and is relatively inexpensive, cross-reactions between the antibodies can occur. Note that multiplex real-time PCR assay can be also used for discriminating *Legionella* spp., *L. pneumophila*, and *L. pneumophila* sg. 1, but this technique could be expensive and, therefore, scarcely utilized in routine situations (Mérault et al., 2011). The use of FT-IR spectroscopy would solve the abovementioned issues related to routine laboratory requirements and the expected coming increase in the number of screened samples. The technique is more user friendly and less time consuming than PCR assays. On the other hand, matrix assisted laser desorption ionization time of flight mass spectrometry (MALDI-TOF-MS) is very accurate for the identification of *Legionella* at species level (Dilger et al., 2016), but shows difficulties in serotyping *L. pneumophila* (Kyritsi et al., 2020). Note that while MALDI-TOF-MS microbial identification is linked to its capability to profiling ribosomal peptides (Campos Braga et al., 2013), direct analysis in real time mass spectrometry captures the lipopolysaccharides (LPS) spectral signatures of the outer membrane of *L. pneumophila* serogroups (Tata et al., 2022). In the same vein, FT-IR reveals the spectral variability of LPS in the different *L. pneumophila* serogroups (Pascale et al., 2022). While FT-IR’s partial capability of identifying each serogroup from 2 to 15, which could help tracing the sources of infections or contaminations (Pascale et al., 2022), the present study sets up a robust method that addresses the requirements of the European regulation (Directive EU 2020/2184 of the european parliament and the council of 16 December 2020 on the quality of water intended for human consumption, 2020) and Italian national guidelines (Linee Guida per la Prevenzione ed. il Controllo della Legionellosi., 2015).

Note that one of the limitations that slows down the translation of non-targeted methods into official and routine analyses is the lack of proper intra-and inter-laboratory validation (Cavanna et al., 2018; Woolman et al., 2021). One of the caveats of non-targeted methods is their strong dependence on statistical correlation of spectral data with the ground truth information enclosed in the training set. As recommended by the sole non-targeted methods guidelines (USP Pharmacopeia, 2018), the robustness of the method must be established in terms of accuracy, sensitivity and specificity and then monitored with an extended validation approach. For this reason, two laboratories successfully challenged the novel non-targeted approach for *Legionella* with multiple sets of samples from a variety of culture collections at different culturing conditions and over the time-span of 1 year. Specifically, the method was tested by evaluating its robustness with multiple operators and IRBT instruments, changing the procedures to produce the solid culture medium (in-house or commercial), and the culture conditions (incubation with CO_2_ 2.5% or in atmospheric air). Finally, the validation was performed over a long time-frame, using *Legionella* isolated from both Italian thermal and drinking water during routine analyses and *Legionella* from European culture collections. We note that the environmental variability of *L. pneumophila* is a significant issue, and is related to the high recombination rates of this species, which produce phenotype variations (Bernander et al., 2004). However, as the spectral database grows and more spectra can be included in the training set, it is likely the phenotype variations will be accounted for.

Previous studies have already shown the successful discrimination of *Salmonella enterica* serogroups (Cordovana et al., 2022) and (para-) typhoid (Cordovana et al., 2021) by FT-IR spectroscopy and machine learning and the same was recently explored for the serotyping of each of the *L. pneumophila* serogroups and the exploratory differentiation of *L. pneumophila* sg. 1 from sg. 2-15 (Pascale et al., 2022). Note that Pascale et al. fully differentiated by FT-IR only *L. pneumophila* serogroups 1, 7 and 11. Based on this preliminary finding, a comprehensive study will be performed, involving different centers and several isolates (to catch the possible geographical variance), to explore the discrimination power of this technology at single serogroup level. On the contrary, the present study developed and fully validated a method, based on the coupling of FT-IR and machine learning, for the automated differentiation of *L. pneumophila* sg.1 and sg. 2-15 as well as their discrimination from *Legionella* non-*pneumophila*. We definitely established the robustness of this approach and its possible adoption in a routine laboratory as it delivers the mandatory outcomes required by National and International guidelines (Linee Guida per la Prevenzione ed. il Controllo della Legionellosi., 2015). Such innovative platform for microbial differentiation will drive the introduction of a next generation spectroscopy devices, coupled to machine learning, in routine laboratories in a fully accredited manner. While multiple explorations of FT-IR in applied microbiology are being continuously reported, the multi centric validation described here will open new avenues through the full accreditation of the method and the consequent incorporation in routine laboratories.

## 5. Conclusion

This study established the performances of a FT-IR-based method for the discrimination of *L. pneumophila* sg. 1, *L. pneumophila* sg. 2-15, and *Legionella* non-*pneumophila*. The method achieved very high overall accuracy in three different independent validation stages and addressed late-stage concerns related to encountering rare *L.* non-*pneumophila*. This multi-centric study opens up new avenues for the routine clinical diagnostics and environmental surveillance of *L. pneumophila*, as it demonstrates a reliable and fast screening method for sg. 1 and the correct classification of *L.* non-*pneumophila* not yet included in the training set of the machine learning classifier. Moreover, the simple extraction procedure with ethanol, the short deposition time, and the rapidity of data acquisition facilitates the easy implementation of this FT-IR spectroscopy technique into routine laboratories as a microbial screening technology that could easily overcome the shortcomings of the conventional techniques. This discrimination method, once made routine and linked to alert systems, should contribute to epidemiological surveillance of *Legionella* diseases, early detection of clusters of *L. pneumophila* sg. 1, and rapid warning of potential outbreaks. Further investigations are being undertaken for the generation of a classifier able to distinguish *Legionella* spp. from other non-*Legionella* bacteria, with similar morphology, that cohabit in water. Research is still ongoing.

## Data availability statement

The original contributions presented in the study are included in the article/Supplementary material, further inquiries can be directed to the corresponding author.

## Author contributions

RP, LB, and SB designed and supervised the study. AT, MC, and FM wrote the original draft, reviewed, and edited the manuscript. FM cultured the samples. ATa, ATi, and FM carried out the FT-IR experiments. MC and AM built-up and validated the classifier. All authors contributed to the article and approved the submitted version.

## Conflict of interest

MC was employed by Bruker Daltonic GmbH (the manufacturer of IR-Biotyper R).

The remaining authors declare that the research was conducted in the absence of any commercial or financial relationships that could be construed as a potential conflict of interest.

## Publisher’s note

All claims expressed in this article are solely those of the authors and do not necessarily represent those of their affiliated organizations, or those of the publisher, the editors and the reviewers. Any product that may be evaluated in this article, or claim that may be made by its manufacturer, is not guaranteed or endorsed by the publisher.
