# Supplementary material for: A multi-center validation study on the discrimination of Legionella pneumophila sg.1, Legionella pneumophila sg. 2-15 and Legionella non-pneumophila isolates from water by FT-IR spectroscopy
Source: Front Microbiol. 2023 Apr 13;14:1150942. doi: 10.3389/fmicb.2023.1150942 (PMC10133462; doi:10.3389/fmicb.2023.1150942)
Supplement: Supplementary file 1 [file Data_Sheet_1.docx]

**A multi-center validation study on the discrimination of *Legionella pneumophila* sg.1 and sg. 2-15 and *Legionella* non-*pneumophila* isolates from water by FT-IR spectroscopy.**

**Alessandra Tata^1#^, Filippo Marzoli^2#^, Miriam Cordovana^3^, Alessia Tiengo^4^, Carmela Zacometti^1^, Andrea Massaro^1^, Lisa Barco^4^, Simone Belluco^2^, Roberto Piro^1^.**

^1^Istituto Zooprofilattico Sperimentale delle Venezie, Laboratorio di Chimica Sperimentale, Vicenza, Italy.

^2^Department of Food Safety, Istituto Zooprofilattico Sperimentale delle Venezie, Legnaro, Italy.

^3^Bruker Daltonics GmbH & Co. KG, Bremen, Germany.

^4^OIE Italian Reference Laboratory for Salmonella, Istituto Zooprofilattico Sperimentale delle Venezie, Padova, Italy.

*** Correspondence:**Alessandra Tata
[atata@izsvenezie.it](mailto:atata@izsvenezie.it)

^#^ These authors contributed equally to the work.

**SUPPLEMENTARY MATERIAL**

**Table S1. *Legionella* isolates of the training set used to build up the SVM classifier.**

|  | |  |
| --- | --- | --- |
| **Isolates** | **Group** | |
| Lpne_IZSVI 1-3922/1 | *L. pneumophila* sg.1 | |
| Lpne_IZSVI 19-2758/3 | *L. pneumophila* sg.1 | |
| Lpne_IZSVI 21-2755/5 | *L. pneumophila* sg.1 | |
| Lpne_IZSVI 2-2672/2 | *L. pneumophila* sg.1 | |
| Lpne_IZSVI 2749/1 | *L. pneumophila* sg. 2-15 | |
| Lpne_IZSVI 2840/5 | *L. pneumophila* sg. 2-15 | |
| Lpne_IZSVI 8-2692/5 | *L. pneumophila* sg. 2-15 | |
| Lpne_IZSVI 9-2692/4 | *L. pneumophila* sg. 2-15 | |
| Lpne_IZSVI L. anisa | L. non-*pneumophila* | |
| Lpne_IZSVI L. bozemanii | L. non-*pneumophila* | |
| Lpne_IZSVI L. dumoffii | L. non-*pneumophila* | |
| Lpne_IZSVI L. feeleii | L. non-*pneumophila* | |
| Lpne_IZSVI L. gormani | L. non-*pneumophila* | |
| Lpne_IZSVI L. jordanis | L. non-*pneumophila* | |
| Lpne_IZSVI L. longbeacheae | L. non-*pneumophila* | |
| Lpne_IZSVI L. micdadei | L. non-*pneumophila* | |
| Lpne_IZSVI L. rubrilucens | L. non-*pneumophila* | |
| Lpne_IZSVI L. taurinensis | L. non-*pneumophila* | |
|  | |  |

**Table S2. Results of evaluation of the SVM classifier on the withheld test set (n=64). A “green score” means that the result is highly reliable. A “yellow score” indicates that the result of the prediction is moderately reliable. A “red score” value means that the prediction cannot be considered reliable, as the isolate spectra are located in the spectral space far from the samples included in the training set, and therefore, they could either not belong to any known class included in the training set, or the sample shows a very high technical or biological variance. All the isolates were correctly classified.**

| **Samples** | **Actual** | **Predicted** |
| --- | --- | --- |
| Lpne_1-IZSVI 2800/4 | *L. pneumophila* sg. 1 | *L. pneumophila* sg. 1 |
| Lpne_IZSVI 1-3332 | *L. pneumophila* sg. 1 | *L. pneumophila* sg. 1 |
| Lpne_IZSVI 1-3922/1 | *L. pneumophila* sg. 1 | *L. pneumophila* sg. 1 |
| Lpne_IZSVI 10-2692/3 | *L. pneumophila* sg. 1 | *L. pneumophila* sg. 1 |
| Lpne_IZSVI 10-2902 | *L. pneumophila* sg. 1 | *L. pneumophila* sg. 1 |
| Lpne_IZSVI 10-3323/2 | *L.* non- *pneumophila* | *L.* non- *pneumophila* |
| Lpne_IZSVI 11-2692/2 | *L. pneumophila* sg. 1 | *L. pneumophila* sg. 1 |
| Lpne_IZSVI 11-2913/2 | *L. pneumophila* sg. 1 | *L. pneumophila* sg. 1 |
| Lpne_IZSVI 11-3303/9 | *L.* non- *pneumophila* | *L.* non- *pneumophila* |
| Lpne_IZSVI 12-2718/2 | *L. pneumophila* sg. 1 | *L. pneumophila* sg. 1 |
| Lpne_IZSVI 12-2913/1 | *L. pneumophila* sg. 1 | *L. pneumophila* sg. 1 |
| Lpne_IZSVI 13-2672/1 | *L. pneumophila* sg. 2-15 | *L. pneumophila* sg. 2-15 |
| Lpne_IZSVI 13-2755/4 | *L. pneumophila* sg. 2-15 | *L. pneumophila* sg. 2-15 |
| Lpne_IZSVI 14-2710/3 | *L. pneumophila* sg. 2-15 | *L. pneumophila* sg. 2-15 |
| Lpne_IZSVI 14-2755/1 | *L. pneumophila* sg. 2-15 | *L. pneumophila* sg. 2-15 |
| Lpne_IZSVI 15-2710/2 | *L. pneumophila* sg. 2-15 | *L. pneumophila* sg. 2-15 |
| Lpne_IZSVI 15-2749/3 | *L. pneumophila* sg. 2-15 | *L. pneumophila* sg. 2-15 |
| Lpne_IZSVI 16-2710/1 | *L. pneumophila* sg. 2-15 | *L. pneumophila* sg. 2-15 |
| Lpne_IZSVI 16-2749/1 | *L. pneumophila* sg. 2-15 | *L. pneumophila* sg. 2-15 |
| Lpne_IZSVI 17-2672/5 | *L. pneumophila* sg. 2-15 | *L. pneumophila* sg. 2-15 |
| Lpne_IZSVI 17-2745/6 | *L. pneumophila* sg. 2-15 | *L. pneumophila* sg. 2-15 |
| Lpne_IZSVI 18-2745/2 | *L. pneumophila* sg. 2-15 | *L. pneumophila* sg. 1 |
| Lpne_IZSVI 18-2969/2 | *L. pneumophila* sg. 2-15 | *L. pneumophila* sg. 2-15 |
| Lpne_IZSVI 19-2745/1 | *L. pneumophila* sg. 2-15 | *L. pneumophila* sg. 2-15 |
| Lpne_IZSVI 19-2758/3 | *L. pneumophila* sg. 2-15 | *L. pneumophila* sg. 2-15 |
| Lpne_IZSVI 2-2672/2 | *L. pneumophila* sg. 1 | *L. pneumophila* sg. 1 |
| Lpne_IZSVI 2-2769/1 | *L. pneumophila* sg. 1 | *L. pneumophila* sg. 1 |
| Lpne_IZSVI 2-3247 | *L. pneumophila* sg. 1 | *L. pneumophila* sg. 1 |
| Lpne_IZSVI 20-2755/6 | *L. pneumophila* sg. 2-15 | *L. pneumophila* sg. 2-15 |
| Lpne_IZSVI 20-2841/2 | *L. pneumophila* sg. 2-15 | *L. pneumophila* sg. 2-15 |
| Lpne_IZSVI 21-2755/5 | *L. pneumophila* sg. 2-15 | *L. pneumophila* sg. 2-15 |
| Lpne_IZSVI 21-2822/1 | *L. pneumophila* sg. 2-15 | *L. pneumophila* sg. 2-15 |
| Lpne_IZSVI 22-2672/2 | *L. pneumophila* sg. 1 | *L. pneumophila* sg. 1 |
| Lpne_IZSVI 22-2840/5 | *L. pneumophila* sg. 2-15 | *L. pneumophila* sg. 2-15 |
| Lpne_IZSVI 22-Legio MR lab | *L. pneumophila* sg. 2-15 | *L. pneumophila* sg. 2-15 |
| Lpne_IZSVI 23-2672/2 | *L. pneumophila* sg. 2-15 | *L. pneumophila* sg. 2-15 |
| Lpne_IZSVI 23-2692/4 | *L. pneumophila* sg. 1 | *L. pneumophila* sg. 1 |
| Lpne_IZSVI 23-2840/6 | *L. pneumophila* sg. 2-15 | *L. pneumophila* sg. 2-15 |
| Lpne_IZSVI 24-2672/3 | *L.* non- *pneumophila* | *L.* non- *pneumophila* |
| Lpne_IZSVI 24-2692/3 | *L. pneumophila* sg. 1 | *L. pneumophila* sg. 1 |
| Lpne_IZSVI 24-2857/4 | *L. pneumophila* sg. 2-15 | *L. pneumophila* sg. 2-15 |
| Lpne_IZSVI 25-2770 | *L.* non- *pneumophila* | *L.* non- *pneumophila* |
| Lpne_IZSVI 25-2857/3 | *L. pneumophila* sg. 2-15 | *L. pneumophila* sg. 2-15 |
| Lpne_IZSVI 26-2758/1 | *L.* non- *pneumophila* | *L.* non- *pneumophila* |
| Lpne_IZSVI 26-2857/2 | *L. pneumophila* sg. 2-15 | *L. pneumophila* sg. 2-15 |

**Table S3. Results of inter-laboratory evaluation of the SVM classifier on Legionella isolates from different geographical origins (n=129). A “green score” means that the result is highly reliable. A “yellow score” indicates that the result of the prediction is moderately reliable. A “red score” value means that the prediction cannot be considered reliable, as the isolate spectra are located in the spectral space far from the samples included in the training set, and therefore, they could either not belong to any known class included in the training set, or the sample shows a very high technical or biological variance. The star * indicates incorrect classification with green outcome. Note that no red outcomes were obtained.**

| **Samples** | **Actual** | **Predicted** |
| --- | --- | --- |
| CCUG 13395 (6) | *L. pneumophila* sg. 1 | *L. pneumophila sg. 1* |
| **CCUG 30660 (6)** | *L. pneumophila* sg. 2-15 | *L.* non- *pneumophila** |
| CCUG 61603 (6) | *L. pneumophila* sg. 2-15 | *L. pneumophila* sg. 2-15 |
| CIP 103857 (18) | *L. pneumophila* sg. 2-15 | *L. pneumophila* sg. 2-15 |
| CIP 103859 (18) | *L. pneumophila* sg. 2-15 | *L. pneumophila* sg. 2-15 |
| CIP 103860 (18) | *L. pneumophila* sg. 2-15 | *L. pneumophila* sg. 2-15 |
| **CIP 103861 (18)** | *L. pneumophila* sg. 2-15 | *L. pneumophila* sg. 1* |
| CIP 103862 (18) | *L. pneumophila* sg. 2-15 | *L. pneumophila* sg. 2-15 |
| CIP 103863 (18) | *L. pneumophila* sg. 2-15 | *L. pneumophila* sg. 2-15 |
| CIP 103864 (18) | *L. pneumophila* sg. 2-15 | *L. pneumophila* sg. 2-15 |
| CIP 103865 (18) | *L. pneumophila* sg. 2-15 | *L. pneumophila* sg. 2-15 |
| CIP 103866 (18) | *L. pneumophila* sg. 2-15 | *L. pneumophila* sg. 2-15 |
| CIP 103867 (18) | *L. pneumophila* sg. 2-15 | *L. pneumophila* sg. 2-15 |
| CIP 103868 (18) | *L. pneumophila* sg. 2-15 | *L. pneumophila* sg. 2-15 |
| CIP 103869 (18) | *L. pneumophila* sg. 2-15 | *L. pneumophila* sg. 2-15 |
| CVUAS 3932 (69) | *L. pneumophila* sg. 2-15 | *L. pneumophila* sg. 2-15 |
| CVUAS 4013 (69) | *L. pneumophila* sg. 2-15 | *L. pneumophila* sg. 2-15 |
| CVUAS 4014 (69) | *L. pneumophila* sg. 2-15 | *L. pneumophila* sg. 2-15 |
| CVUAS 4122 (63) | *L. pneumophila* sg. 1 | *L. pneumophila* sg. 1 |
| CVUAS 4126 (36) | *L. pneumophila* sg. 1 | *L. pneumophila* sg. 1 |
| CVUAS 4132 (36) | *L. pneumophila* sg. 1 | *L. pneumophila* sg. 1 |
| CVUAS 4133 (63) | *L. pneumophila* sg. 1 | *L. pneumophila* sg. 1 |
| CVUAS 4136 (63) | *L. pneumophila* sg. 1 | *L. pneumophila* sg. 1 |
| CVUAS 4137 (36) | *L. pneumophila* sg. 1 | *L. pneumophila* sg. 1 |
| CVUAS 4138 (36) | *L. pneumophila* sg. 1 | *L. pneumophila* sg. 1 |
| CVUAS 4140 (63) | *L. pneumophila* sg. 1 | *L. pneumophila* sg. 1 |
| CVUAS 4141 (36) | *L. pneumophila* sg. 1 | *L. pneumophila* sg. 1 |
| CVUAS 4142 (63) | *L. pneumophila* sg. 1 | *L. pneumophila* sg. 1 |
| CVUAS 4255 (48) | *L. pneumophila* sg. 2-15 | *L. pneumophila* sg. 2-15 |
| CVUAS 4325 (42) | *L. pneumophila* sg. 2-15 | *L. pneumophila* sg. 2-15 |
| CVUAS 4326 (69) | *L. pneumophila* sg. 2-15 | *L. pneumophila* sg. 2-15 |
| CVUAS 4327 (36) | *L. pneumophila* sg. 2-15 | *L. pneumophila* sg. 2-15 |
| CVUAS 4375 (48) | *L. pneumophila* sg. 2-15 | *L. pneumophila* sg. 2-15 |
| CVUAS 4376 (36) | *L. pneumophila* sg. 2-15 | *L. pneumophila* sg. 2-15 |
| CVUAS 4656 (63) | *L. pneumophila* sg. 2-15 | *L. pneumophila* sg. 2-15 |
| CVUAS 4805 (42) | *L. pneumophila* sg. 2-15 | *L. pneumophila* sg. 2-15 |
| CVUAS 5308 (75) | *L. pneumophila* sg. 2-15 | *L. pneumophila* sg. 2-15 |
| CVUAS 5333 (63) | *L. pneumophila* sg. 1 | *L. pneumophila* sg. 1 |
| CVUAS 5747 (63) | *L. pneumophila* sg. 1 | *L. pneumophila* sg. 1 |
| CVUAS 5748 (36) | *L. pneumophila* sg. 1 | *L. pneumophila* sg. 1 |
| CVUAS 5749 (36) | *L. pneumophila* sg. 1 | *L. pneumophila* sg. 1 |
| CVUAS 5750 (63) | *L. pneumophila* sg. 1 | *L. pneumophila* sg. 1 |
| CVUAS 5751 (36) | *L. pneumophila* sg. 1 | *L. pneumophila* sg. 1 |
| CVUAS 5752 (63) | *L. pneumophila* sg. 1 | *L. pneumophila* sg. 1 |
| CVUAS 5753 (42) | *L. pneumophila* sg. 2-15 | *L. pneumophila* sg. 2-15 |
| CVUAS 5754 (42) | *L. pneumophila* sg. 2-15 | *L. pneumophila* sg. 2-15 |
| CVUAS 5755 (42) | *L. pneumophila* sg. 2-15 | *L. pneumophila* sg. 2-15 |
| CVUAS 5850 (42) | *L. pneumophila* sg. 2-15 | *L. pneumophila* sg. 2-15 |
| CVUAS 5852 (69) | *L. pneumophila* sg. 2-15 | *L. pneumophila* sg. 2-15 |
| CVUAS 5887 (36) | *L. pneumophila* sg. 1 | *L. pneumophila* sg. 1 |
| CVUAS 6110 (42) | *L. pneumophila* sg. 2-15 | *L. pneumophila* sg. 2-15 |
| CVUAS 7552.2 (42) | *L. pneumophila* sg. 2-15 | *L. pneumophila* sg. 2-15 |
| CVUAS 7553.2 (36) | *L. pneumophila* sg. 1 | *L. pneumophila* sg. 1 |
| DSM 10572 | *L. longbeachae* | *L.* non- *pneumophila* |
| DSM 109129 | *L. bozemanii* | *L.* non- *pneumophila* |
| DSM 17627T | *L. anisa* | *L.* non- *pneumophila* |
| DSM 25284 | *L. micdadei* | *L.* non- *pneumophila* |
| DSM 25315 | *L. longbeachae* | *L.* non- *pneumophila* |
| DSM 25316 | *L. feeleii* | *L.* non- *pneumophila* |
| DSM 7513 (63) | *L. pneumophila* sg. 1 | *L. pneumophila* sg. 1 |
| DSM 7514 (63) | *L. pneumophila* sg. 2-15 | *L. pneumophila* sg. 2-15 |
| DSM 7515 (63) | *L. pneumophila* sg. 2-15 | *L. pneumophila* sg. 2-15 |
| NCTC 12821 (6) | *L. pneumophila* sg. 1 | *L. pneumophila* sg. 1 |
| Ried 004 (6) | *L. pneumophila* sg. 2-15 | *L. pneumophila* sg. 2-15 |
| Ried 009 (6) | *L. pneumophila* sg. 2-15 | *L. pneumophila* sg. 2-15 |
| Ried 019 (6) | *L. pneumophila* sg. 2-15 | *L. pneumophila* sg. 2-15 |
| Ried_001_KBSR (36) | *L. pneumophila* sg. 2-15 | *L. pneumophila* sg. 2-15 |
| Ried_004_KBSR (36) | *L. pneumophila* sg. 2-15 | *L. pneumophila* sg. 2-15 |
| Ried_006_KBSR (36) | *L. pneumophila* sg. 2-15 | *L. pneumophila* sg. 2-15 |
| Ried_007_KBSR (36) | *L. pneumophila* sg. 1 | *L. pneumophila* sg. 1 |
| Ried_009_KBSR (36) | *L. pneumophila* sg. 2-15 | *L. pneumophila* sg. 2-15 |
| Ried_011_KBSR (42) | *L. pneumophila* sg. 2-15 | *L. pneumophila* sg. 2-15 |
| Ried_012_KBSR (42) | *L. pneumophila* sg. 2-15 | *L. pneumophila* sg. 2-15 |
| Ried_013_KBSR (42) | *L. pneumophila* sg. 2-15 | *L. pneumophila* sg. 2-15 |
| Ried_014_KBSR (42) | *L. pneumophila* sg. 2-15 | *L. pneumophila* sg. 2-15 |
| Ried_015_KBSR (36) | *L. pneumophila* sg. 1 | *L. pneumophila* sg. 1 |
| Ried_016_KBSR (42) | *L. pneumophila* sg. 2-15 | *L. pneumophila* sg. 2-15 |
| Ried_017_KBSR (42) | *L. pneumophila* sg. 2-15 | *L. pneumophila* sg. 2-15 |
| Ried_019_KBSR (36) | *L. pneumophila* sg. 2-15 | *L. pneumophila* sg. 2-15 |
| Ried_020_KBSR (42) | *L. pneumophila* sg. 2-15 | *L. pneumophila* sg. 2-15 |
| Ried_023_KBSR (42) | *L. pneumophila* sg. 2-15 | *L. pneumophila* sg. 2-15 |
| Ried_030_KBSR (36) | *L. pneumophila* sg. 2-15 | *L. pneumophila* sg. 2-15 |
| Ried_032_KBSR (36) | *L. pneumophila* sg. 2-15 | *L. pneumophila* sg. 2-15 |
| Ried_035_KBSR (42) | *L. pneumophila* sg. 2-15 | *L. pneumophila* sg. 2-15 |
| Ried_045_KBSR (42) | *L. pneumophila* sg. 2-15 | *L. pneumophila* sg. 2-15 |
| Ried_047_KBSR (36) | *L. pneumophila* sg. 1 | *L. pneumophila* sg. 1 |
| Ried_048_KBSR (36) | *L. pneumophila* sg. 1 | *L. pneumophila* sg. 1 |
| Ried_049_KBSR (36) | *L. pneumophila* sg. 2-15 | *L. pneumophila* sg. 2-15 |
| Ried_050_KBSR (36) | *L. pneumophila* sg. 1 | *L. pneumophila* sg. 1 |
| Ried_052_KBSR (36) | *L. pneumophila* sg. 2-15 | *L. pneumophila* sg. 2-15 |
| Ried_058_KBSR (36) | *L. pneumophila* sg. 2-15 | *L. pneumophila* sg. 2-15 |
| Ried_061_KBSR (42) | *L. pneumophila* sg. 2-15 | *L. pneumophila* sg. 2-15 |
| Ried_068_KBSR (36) | *L. pneumophila* sg. 1 | *L. pneumophila* sg. 1 |
| Ried_069_KBSR (36) | *L. pneumophila* sg. 1 | *L. pneumophila* sg. 1 |
| Ried_122_KBSR (36) | *L. pneumophila* sg. 1 | *L. pneumophila* sg. 1 |
| Ried_124_KBSR (36) | *L. pneumophila* sg. 1 | *L. pneumophila* sg. 1 |
| Ried_126_KBSR (36) | *L. pneumophila* sg. 1 | *L. pneumophila* sg. 1 |
| Ried_147_KBSR (36) | *L. pneumophila* sg. 1 | *L. pneumophila* sg. 1 |
| Ried_149_KBSR (36) | *L. pneumophila* sg. 1 | *L. pneumophila* sg. 1 |
| Ried_151_KBSR (36) | *L. pneumophila* sg. 1 | *L. pneumophila* sg. 1 |
| Ried_158_KBSR (36) | *L. pneumophila* sg. 1 | *L. pneumophila* sg. 1 |
| Ried_H001_KBSR (36) | *L. pneumophila* sg. 1 | *L. pneumophila* sg. 1 |
| Ried_H005_KBSR (36) | *L. pneumophila* sg. 1 | *L. pneumophila* sg. 1 |
| Ried_H006_KBSR (42) | *L. pneumophila* sg. 2-15 | *L. pneumophila* sg. 2-15 |
| Ried_H009_KBSR (42) | *L. pneumophila* sg. 2-15 | *L. pneumophila* sg. 2-15 |
| Ried_H010_KBSR (36) | *L. pneumophila* sg. 1 | *L. pneumophila* sg. 1 |
| Ried_H011_KBSR (36) | *L. pneumophila* sg. 1 | *L. pneumophila* sg. 1 |
| Ried_H012_KBSR (36) | *L. pneumophila* sg. 1 | *L. pneumophila* sg. 1 |
| Ried_Leg_01 (36) | *L. pneumophila* sg. 1 | *L. pneumophila* sg. 1 |
| Ried_Leg_02 (42) | *L. pneumophila* sg. 2-15 | *L. pneumophila* sg. 2-15 |
| Ried_Leg_03 (42) | *L. pneumophila* sg. 2-15 | *L. pneumophila* sg. 2-15 |
| Ried_Leg_04 (36) | *L. pneumophila* sg. 1 | *L. pneumophila* sg. 1 |
| Ried_Leg_06 (36) | *L. pneumophila* sg. 1 | *L. pneumophila* sg. 1 |
| Ried_Leg_07 (42) | *L. pneumophila* sg. 2-15 | *L. pneumophila* sg. 2-15 |
| 16640T DSM (6) | *L. micadadei* | *L.* non- *pneumophila* |
| 16641T DSM (6) | *L. gormanii* | *L.* non- *pneumophila* |
| 170613_13 (6) | *L. anisa* | *L.* non- *pneumophila* |
| 21897T DSM (6) | *L. tauriniensis* | *L.* non- *pneumophila* |
| 25283 DSM (6) | *L. micadadei* | *L.* non- *pneumophila* |
| 25296T DSM (6) | *L. gormanii* | *L.* non- *pneumophila* |
| 28612 CCUG (6) | *L. longbeachae* | *L.* non- *pneumophila* |
| 31569 CCUG (6) | *L. bozemanii* | *L.* non- *pneumophila* |
| 49017 CCUG (6) | *L. bozemanii* | *L.* non- *pneumophila* |
| 64423 CCUG (6) | *L. longbeachae* | *L.* non- *pneumophila* |
| *L. feelei* DSM 17645 (6) | *L.feelei* | *L.* non- *pneumophila* |
| *L .jordanis* DSM 19212T (6) | *L. jordanis* | *L.* non- *pneumophila* |
| *L. longbeachae* DSM 10572 (6) | *L. longbeachae* | *L.* non- *pneumophila* |
| 109128 DSM (3) | *L. longbeachae* | *L.* non- *pneumophila* |
| 17625T DSM (3) | *L. dumoffii* | *L.* non- *pneumophila* |
| 48836 CCUG (3) | *L. bozemanii* | *L.* non- *pneumophila* |
| *L. micdadei* DSM 25284 (3) | *L. micadadei* | *L.* non- *pneumophila* |

**Table S4. Validation of the SVM classifier on unusual *L.* *non-pneumophila* isolates (n=33). A “green score” means that the result is highly reliable. A “yellow score” indicates that the result of the prediction is moderately reliable. A “red score” value means that the prediction cannot be considered reliable, as the isolate spectra are located in the spectral space far from the samples included in the training set, and therefore, they could either not belong to any known class included in the training set, or the sample shows a very high technical or biological variance. The star * indicates incorrect classification with green outcome.**

| **Samples** | **Actual** | **Predicted** |
| --- | --- | --- |
| DSM 105266T | *L. fairfieldensis* | *L.* non- *pneumophila* |
| DSM 21233T | *L. gratiana* | *L.* non- *pneumophila* |
| DSM 19215 | *L. jamestownensis* | *L.* non- *pneumophila* |
| DSM 19556 | *L. lansingensis* | *L.* non- *pneumophila* |
| DSM 21234T | *L. londiniensis* | *L.* non- *pneumophila* |
| DSM 24804 | *L. massiliensis* | *L.* non- *pneumophila* |
| DSM 24727 | *L. nagasakiensis* | *L.* non- *pneumophila* |
| DSM 21805T | *L. nautarum* | *L.* non- *pneumophila* |
| DSM 105104T | *L. norrlandica* | *L.* non- *pneumophila* |
| DSM 21215T-a | *L. oakridgensis* | ***L. pneumophila sg. 2– 15**** |
| DSM 19216 | *L. parisiensis* | *L.* non- *pneumophila* |
| CIP 105271T | *L. quateirensis* | *L.* non- *pneumophila* |
| DSM 109131 | *L. quinlivani* | *L.* non- *pneumophila* |
| DSM 21216T | *L. quinlivani* | *L.* non- *pneumophila* |
| DSM 19231 | *L. sainthelenensis* | *L.* non- *pneumophila* |
| DSM 25322 | *L. sainthelenensis* | *L.* non- *pneumophila* |
| DSM 23075 | *L. santacrucis* | *L.* non- *pneumophila* |
| DSM 23076T | *L. steigerwaltii* | *L.* non- *pneumophila* |
| DSM 19246 | *L. tucsonensis* | *L.* non- *pneumophila* |
| DSM 21896T | *L. wadsworthii* | *L.* non- *pneumophila* |
| DSM 21908T | *L. waltersii* | *L.* non- *pneumophila* |
| DSM 21907 | *L. worsleiensis* | *L.* non- *pneumophila* |
| DSM 19232 | *L. birminghamensis* | *L.* non- *pneumophila* |
| DSM 19236T | *L. brunensis* | *L.* non- *pneumophila* |
| DSM 22853 | *L. busanensis* | *L.* non- *pneumophila* |
| DSM 25049T | *L. cardiaca* | ***L. pneumophila* sg. 2– 15*** |
| DSM 19213 | *L. cherrii* | *L.* non- *pneumophila* |
| DSM 19233 | *L. cincinnatiensis* | *L.* non- *pneumophila* |
| DSM 17644T | *L. erythra* | *L.* non- *pneumophila* |
| DSM 19889T | *L. fallonii* | *L.* non- *pneumophila* |
| DSM 21218T | *L. gresisliensis* | *L.* non- *pneumophila* |
| DSM 25323 | *L. hackeliae* | *L.* non- *pneumophila* |
| DSM 19214 | *L. hackeliae* | *L.* non- *pneumophila* |
